# Genetic diversity and connectivity within *Mytilus* spp. in the subarctic and Arctic

**DOI:** 10.1111/eva.12415

**Published:** 2016-10-26

**Authors:** Sofie Smedegaard Mathiesen, Jakob Thyrring, Jakob Hemmer‐Hansen, Jørgen Berge, Alexey Sukhotin, Peter Leopold, Michaël Bekaert, Mikael Kristian Sejr, Einar Eg Nielsen

**Affiliations:** ^1^ Department of BioscienceArctic Research CentreAarhus UniversityAarhus CDenmark; ^2^ Section for Marine Living ResourcesNational Institute of Aquatic ResourcesTechnical University of DenmarkSilkeborgDenmark; ^3^ Faculty of Biosciences, Fisheries and EconomicsUiT The Arctic University of NorwayTromsøNorway; ^4^ The University Centre in SvalbardLongyearbyenNorway; ^5^ White Sea Biological StationZoological Institute of Russian Academy of SciencesSt. PetersburgRussia; ^6^ Invertebrate Zoology DepartmentSt. Petersburg State UniversitySt. PetersburgRussia; ^7^ Institute of AquacultureUniversity of StirlingStirlingUK

**Keywords:** arctic fauna, bivalves, climate change, glacial refugium, hybrid zone, *Mytilus edulis*, population structure, SNPs

## Abstract

Climate changes in the Arctic are predicted to alter distributions of marine species. However, such changes are difficult to quantify because information on present species distribution and the genetic variation within species is lacking or poorly examined. Blue mussels, *Mytilus* spp., are ecosystem engineers in the coastal zone globally. To improve knowledge of distribution and genetic structure of the *Mytilus edulis* complex in the Arctic, we analyzed 81 SNPs in 534 *Mytilus* spp. individuals sampled at 13 sites to provide baseline data for distribution and genetic variation of *Mytilus* mussels in the European Arctic. *Mytilus edulis* was the most abundant species found with a clear genetic split between populations in Greenland and the Eastern Atlantic. Surprisingly, analyses revealed the presence of *Mytilus trossulus* in high Arctic NW Greenland (77°N) and *Mytilus galloprovincialis* or their hybrids in SW Greenland, Svalbard, and the Pechora Sea. Furthermore, a high degree of hybridization and introgression between species was observed. Our study highlights the importance of distinguishing between congener species, which can display local adaptation and suggests that information on dispersal routes and barriers is essential for accurate predictions of regional susceptibility to range expansions or invasions of boreal species in the Arctic.

## 1 Introduction

Nowhere else on Earth is the impact of climate change expected to be more severe than in the Arctic. Temperatures in the Arctic are estimated to increase by 4–7°C over the next century, with wide‐ranging effects on Arctic species (ACIA 2004; IPCC 2014). This have caused shifts in species' abundances and distributions over the last decades (IPCC 2014; Poloczanska et al., 2013), and future temperature increases are believed to move species distribution limits toward the poles (ACIA 2004). Such effects, however, are nearly impossible to monitor and understand without proper baseline studies of the genetic variation within and between species (Brodersen & Seehausen, 2014). Almost all species investigated, including Arctic, have revealed genetically discrete populations that inhabit a specific subset of the species geographical and environmental range. These populations can exhibit different adaptations and tolerance limits to specific environments (Limborg et al., 2012; Nielsen, Hemmer‐Hansen, Larsen, & Bekkevold, 2009; Thyrring, Rysgaard, Blicher, & Sejr, 2015), which make it important to know their current distribution and the connectivity between populations and the processes governing the distribution of genetic variation. Through genetic analysis, it is possible to determine the level of genetic variability within both threatened and newly established populations, the origin of migrating individuals, direction of gene flow, and possible adaptive evolutionary changes associated with climate change (Brodersen & Seehausen, 2014; Hansen, Olivieri, Waller, & Nielsen, 2012; Laikre, Schwartz, Waples, & Ryman, 2010). All key factors needed to make predictions for the likely impact of climate change.

Bivalves of the genus *Mytilus* are frequently used as environmental indicators, as they are semi‐sessile, have a relatively long life span, and are widely distributed in coastal regions in both Northern Hemisphere and Southern Hemisphere (Goldberg, 1986; Gosling, 2003; Rainbow, 1995; Thyrring, Juhl, Holmstrup, Blicher, & Sejr, 2015). *Mytilus* spp. are commercially and ecologically important species and often a dominant part of the intertidal and shallow subtidal fauna. Therefore, numerous studies of their responses to various stressors (e.g., temperature, salinity, pollutants) have been performed (Gosling, 2003; Jones, Lima, & Wethey, 2010; Mubiana, Qadah, Meys, & Blust, 2005; Søndergaard, Asmund, Johansen, & Riget, 2011; Wanamaker et al., 2007). Furthermore, *Mytilus* spp. have already demonstrated adaptations to different environments (Blicher, Sejr, & Høgslund, 2013; Thyrring, Rysgaard, et al., 2015) and a shift in their southern geographical range caused by increasing temperatures (Jones et al., 2010), making them an excellent model for inferring how species distributions might change in response to climate change. Additionally, *Mytilus* spp. have been the subjects of genetic studies for decades as the different species are morphologically difficult to distinguish. Consequently, the population structure of individual *Mytilus* species has been difficult to establish. *Mytilus edulis* L. 1758, *Mytilus trossulus* Gould 1850 and *Mytilus galloprovincialis* Lmk. 1819, all belong to the *M. edulis* species complex and are known to coexist and hybridize with conflicting patterns on the fitness for hybrids. Some studies did not observe any depressed fitness (Doherty, Brophy, & Gosling, 2009; Koehn, 1991; Riginos & Cunningham, 2005; Toro, Thompson, & Innes, 2006), while others (Gardner & Thompson, 2001; Toro, Innes, & Thompson, 2004; Toro, Thompson, & Innes, 2002; Tremblay & Landry, 2016) found a difference in fitness between parental types and hybrids and backcrosses. These findings and numerous studies on introgression between them (Fraïsse, Belkhir, Welch, & Bierne, 2016; Roux et al., 2014) have challenged the isolation species concept (White, 1978); however, they are generally considered to be different species, as they remain ecological distinct despite semipermeable barriers for gene flow and introgression (Bierne, Borsa, et al., 2003; Fraïsse, Roux, Welch, & Bierne, 2014; Saarman & Pogson, 2015). *Mytilus trossulus* is thought to have invaded the Arctic Ocean from the Pacific Ocean around 3.5 million years ago (mya) through the Bering Strait (Rawson & Hilbish, 1995, 1998; Vermeij, 1991). As the Bering Strait closed during glacial periods, allopatric speciation resulted in the evolution of *M. edulis* in the Atlantic*. Mytilus edulis* has since spread to large parts of the Atlantic and due to apparent low gene flow (at least for some loci); *M. edulis* populations on each side of the Atlantic are genetically distinct (Riginos & Henzler, 2008; Riginos, Hickerson, Henzler, & Cunningham, 2004). Speciation between *M. edulis* and *M. galloprovincialis* most likely occurred through allopatric isolation approximately 2.5 mya (Quesada, Gallagher, Skibinski, & Skibinski, 1998; Rawson & Hilbish, 1995, 1998) with secondary contact and introgression occurring around 0.7 mya (Roux et al., 2014). Between interglacial periods 46,000 and 20,000 years ago, *M. trossulus* reinvaded the Arctic Ocean (Rawson & Harper, 2009). From here, it invaded both sides of the Atlantic founding *M. trossulus/M. edulis* hybrid zones along North American and European coasts (Riginos & Cunningham, 2005).

The geographical distribution and genetic population structure of *Mytilus* spp. have been intensively studied in boreal and temperate regions (Bierne, Borsa, et al., 2003; Hilbish, Carson, Plante, Weaver, & Gilg, 2002; Sarver & Foltz, 1993; Väinölä & Strelkov, 2011; Westerbom, Kilpi, & Mustonen, 2002); however, little is known of their distribution and genetic population structure in the Arctic. In the subarctic and Arctic, *M. edulis* is considered the most abundant *Mytilus* species, and it has been recorded in Arctic regions of Russia, along the Norwegian coast, in Iceland and Greenland (Hummel, Colucci, Bogaards, & Strelkov, 2001; Riginos & Henzler, 2008; Sukhotin, Strelkov, Maximovich, & Hummel, 2007; Väinölä & Strelkov, 2011). In Greenland, *Mytilus* spp. populations are found all along the west coast, and southern populations from Tartoq and Narsarsuaq have been shown to be genetically distinct from European *M. edulis* displaying higher resemblance to Canadian and North American *M. edulis* populations (Riginos & Henzler, 2008). Few genetic analyses have been performed on *Mytilus* spp. in Greenland, and most studies have assumed these mussels to be *M. edulis* without genetic verification despite observations of variations in metabolic response to low temperatures between populations from NW and SW Greenland (Thyrring, Rysgaard, et al., 2015). Moreover, in 2004, subtidal *M. edulis* were discovered at the mouth of Isfjorden in Svalbard after 1,000 years of absence (Berge, Johnsen, Nilsen, Gulliksen, & Slagstad, 2005). These mussels were hypothesized to have been transported from Norway by the West Spitsbergen Current in 2002, but their origin has never been confirmed through genetic analysis. *Mytilus trossulus* is less common in Arctic waters. Väinölä and Strelkov (2011) found that *M. trossulus* had a scattered distribution in the White Sea and the Norwegian Sea, and Feder, Norton, and Geller (2003) found live *M. trossulus* in Arctic Alaska in the 1990s. Furthermore, Wenne, Bach, Zbawicka, Strand, and McDonald (2016) has recently reported a NW Greenlandic fjord at Maarmorilik (71°N) to be inhabited by *M. edulis*,* M. trossulus*, and their hybrids. *Mytilus edulis* and *M. trossulus* hybrid zones have also been found and studied on the European and N American Atlantic coasts. Riginos and Cunningham (2005) reviewed the literature on the subject to look at local adaptation and species segregation and found conflicting patterns of species segregation across the Atlantic. In the western Atlantic, *M. trossulus* was found on wave‐exposed open coasts, whereas *M. edulis* appeared to dominate in sheltered areas of low salinity. However, European *M. trossulus* populations from the Baltic Sea appeared to be locally adapted to the prevailing low salinities. The latter is in line with the findings of Wenne et al. (2016), who found a higher prevalence of *M. trossulus* in the inner Maarmorilik fjord compared with the more saline outer fjord. *Mytilus galloprovincialis* normally inhabits warmer waters, but in recent years the species and *M*. *galloprovincialis/M. edulis* hybrids have been observed along the Norwegian coast (Brooks & Farmen, 2013; Riginos & Henzler, 2008). This could be related to human activities like ship traffic in rural areas enabling faster invasion of waters otherwise not directly accessible to them (Anderson, Bilodeau, Gilg, & Hilbish, 2002; Geller, Carlton, & Powers, 1994). Furthermore, it has been demonstrated that *M. galloprovincialis* is capable of tolerating low water temperatures (Inoue et al., 1997), highlighting the potential for this species to occur in the Arctic.

Most studies on *Mytilus* spp. have focused on a few allozymes, mtDNA markers, or microsatellites (Bierne, Daguin, Bonhomme, David, & Borsa, 2003; Brooks & Farmen, 2013; Feder et al., 2003; McDonald, Seed, & Koehn, 1991; Ouagajjou, Presa, Astorga, & Pérez, 2011; Presa, Perez, & Diz, 2002). However, in recent years the use of single nucleotide polymorphisms, SNPs, has become increasingly popular to answer questions about *Mytilus* spp. status, population structure, hybridization, and adaptive variation (Helyar et al., 2011; Saarman & Pogson, 2015; Zbawicka, Drywa, Smietanka, & Wenne, 2012; Zbawicka, Sanko, Strand, & Wenne, 2014).

Utilizing 81 nuclear SNPs, we examined the distribution of *Mytilus* spp. in subarctic and Arctic regions ranging from the eastern Baffin Bay to the Pechora Sea with a special emphasis on the spatiotemporal population structure of *M. edulis*. We further aimed at identifying the source population or populations for the newly discovered *M. edulis* population in Svalbard and whether the observed differences in temperature response found in W Greenland *Mytilus* populations (Thyrring, Rysgaard, et al., 2015) could be caused by genetically based local adaptation.

## 2 Materials and Methods

### 2.1 Study sites and sampling

**Figure 1:**
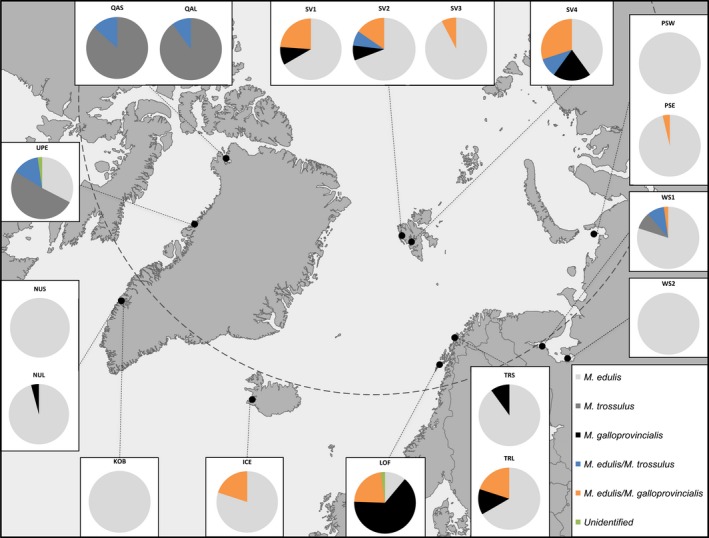
Map showing the different sampling locations and the proportion of three different *Mytilus* species and inferred hybrids at each location. For explanation of sample identification codes, see Table 1. Unidentified individuals denote apparent hybrids of all three *Mytilus* spp.

Nineteen *Mytilus* spp. samples, consisting of 509 individuals in total, were collected from thirteen subarctic and Arctic sites (Table 1 and Fig. 1). Our primary focus was to assure broad geographical coverage and to sample regions where specific hypothesis regarding origin had been generated. The aim was to collect between 30 and 50 individuals from each site. However, as sampling Arctic regions is associated with high logistical costs, we had to rely on already available samples and numbers in some instances. Three samples were collected along the Norwegian NW coast at Tromsø (TRS and TRL) and Lofoten (LOF), and four samples were collected from the Svalbard archipelago (SV1, SV2, SV3, and SV4). Four samples were obtained from the Russian Arctic: two from the White Sea (WS1, WS2) and two from the southeast part of the Barents Sea: Pechora Sea to the east (PSE) and to the west (PSW) of Dolgiy Island. Further, one sample was collected in Iceland: south of Reykjavik (SRI), and six samples were collected in western Greenland: Nuuk (NUS and NUL), Kobbefjord (KOB), Upernavik (UPE), and Qaanaaq (QAS and QAL). From Tromsø, Nuuk, and Qaanaaq, mussels of different size classes were collected as size can be used as a proxy for age class and hence indicate possible short‐term genetic change over time; TRS, NUS, and QAS were smaller mussels in the size range 15–30 mm in length, while TRL, NUL, and QAL being mussels larger than 50 mm.

**Table 1:** Summary of sample information including sampling location and year, sampling code indicating location and possibly size, estimates of expected (*H*_e_), and observed (*H*_o_) heterozygosities and the allelic richness

Country	Location	Latitude	Longitude	Sampling year	Code	*N*	Habitat type	*H*_e_	*H*_o_	Allelic richness
Greenland	Qaanaaq, 15–30 mm	77.4650	−69.2403	2014	QAS	30	Intertidal zone	0.09	0.06	1.40
	Qaanaaq, >50 mm	77.4650	−69.2403	2014	QAL	30	Intertidal zone	0.07	0.04	1.31
	Upernavik	72.7939	−56.1028	2014	UPE	43	Intertidal zone	0.25	0.14	1.73
	Nuuk, 15–30 mm	64.1968	−51.7104	2014	NUS	30	Intertidal zone	0.25	0.23	1.64
	Nuuk, >50 mm			2014	NUL	24	Intertidal zone	0.24	0.21	1.69
	Kobbefjord	64.1367	−51.3909	2014	KOB	30	Intertidal zone	0.25	0.22	1.65
Iceland	Iceland	62.0261	−22.1594	2014	ICE	45	Intertidal zone	0.27	0.27	1.72
Norway	Lofoten	68.3380	13.8780	2014	LOF	45	Intertidal zone	0.26	0.24	1.76
	Tromsø, 15–30 mm	69.8278	18.9226	2014	TRS	10	Intertidal zone	0.27	0.29	1.76
	Tromsø, >50 mm	69.8278	18.9226	2014	TRL	30	Subtidal zone	0.28	0.23	1.64
	Svalbard									
	Kongsfjorden	79.1123	11.1362	2012	SV1	21	Subtidal zone	0.26	0.23	1.73
	Kongsfjorden	79.1123	11.1362	2013	SV2	13	Subtidal zone	0.29	0.24	1.79
	Kongsfjorden	79.1123	11.1362	2014	SV3	13	Subtidal zone	0.26	0.25	1.70
	Adventfjorden	78.2381	15.6026	2014	SV4	10	Subtidal zone	0.29	0.24	1.78
Russia	Pechora Sea, west of Dolgiy Island	69.3563	58.8393	2014	PSW	18	Subtidal zone	0.29	0.29	1.75
	Pechora Sea, east of Dolgiy Island	69.3204	58.7566	2014	PSE	27	Subtidal zone	0.29	0.29	1.77
	White Sea, Kandalaksha Bay	66.3372	33.6494	2014	WS1	45	Subtidal zone	0.32	0.24	1.86
	White Sea, Onega Bay	64.2079	36.6187	2014	WS2	45	Intertidal zone	0.29	0.27	1.77
	Magadan, Okhotsk Sea^a^			2007	MTR	15		0.11	0.01	1.00
Spain	Galician Rías^a^			2009	MGA	10		0.15	0.11	1.27

a These samples were only used for reference i.e. a representative of *Mytilus trossulus* and *Mytilus galloprovincialis*.

Samples were stored at −19°C (TRS, TRL, SRI, KOB, UPE, QAS, and QAL) or in 96% ethanol at 4°C (LOF, SV1, SV2, SV3, SV4, WS1, WS2, PSW, and PSE). Measurements of shell length, width, and height of frozen mussels were conducted at the laboratory facilities at DTU Aqua in Silkeborg, Denmark, whereas the measurements of ethanol preserved specimens were taken at the sampling locations.

Reference samples of *M. trossulus* and *M. galloprovincialis* were provided from the Sea of Okhotsk, Russia, and from around Galician Rías in NW Spain, respectively, to evaluate the species status of the sampled mussels and to identify potential hybrids.

### 2.2 DNA extraction

A minimum of 30 mg (wet weight) of mantle tissue was dissected from each mussel, and DNA was extracted using the Omega EZNA Tissue DNA kit (Omega Bio‐Tek, Norcross, GA, USA) according to the manufacturer's instructions for tissue. DNA content in the extracts was verified on a NanoDrop spectrophotometer (Thermo Scientific, Waltham, MA, USA).

### 2.3 SNP genotyping

SNP genotyping was conducted using the Fluidigm Biomark™ HD System. 96.96 Dynamic Array IFCs were read on a real‐time PCR system after amplification and scored using Fluidigm SNP Genotyping Analysis software. The samples were genotyped for a panel of 96 SNPs: 19 from previous publications on *Mytilus* spp. genetic structure (Zbawicka et al., 2012, 2014) and 77 new SNPs originated from RAD sequencing of genomic *M. edulis* DNA (EBI Sequence Read Archive (SRA) study ERP006912) at the University of Stirling (Table S1).

### 2.4 Summary statistics

Loci with more than 25% missing data across all samples were discarded. Genepop 4.2 (Raymond & Rousset, 1995; Rousset, 2008) was used to test each locus in each sample for departure from Hardy–Weinberg equilibrium (HWE) and linkage disequilibrium (LD) for each locus pair in each sample (10,000 dememorizations, 100 batches, and 5,000 iterations). Within samples, the program diveRsity (Keenan, McGinnity, Cross, Crozier, & Prodohl, 2013) was used to calculate allelic richness and estimate expected (*H*_e_) and observed (*H*_o_) heterozygosities. This was done for both the full data set and for a reduced data set consisting exclusively of inferred *M. edulis* individuals (see explanation in section on *M. edulis* population structure below). Overall and pairwise *F*_ST_ values for all samples were estimated in Genepop 4.2. This initial sorting and discarding of SNP loci resulted in 81 loci being retained for further analyses. As most SNP loci were developed from *M. edulis*, reliable scoring of *M. trossulus* individuals was not possible for three loci (174302_A, 67577_A, and 31051_A), and analyses concerning hybrid identification were performed for 78 SNP loci only.

### 2.5 Identification of hybrids

Based on the generated pairwise *F*_ST_ estimates, the grouping of samples was visualized in a multidimensional scaling plot applying the cmdscale function in R (R Core Team, 2015). Additionally, a principal component analysis scatter plot was created in the R package Adegenet (Jombart, 2008; Jombart & Ahmed, 2011) to illustrate the genetic relationships among individuals across all samples. Structure v2.3.4, utilizing the Bayesian MCMC clustering approach (Pritchard, Stephens, & Donnelly, 2000) was used to visualize species integrity and identify possible hybridization among *Mytilus* spp. using a variable number of predefined clusters (*K*) for grouping individuals. This was also done to positively identify *M. edulis* individuals and subsequently create a reduced data set exclusively aimed at investigating population structure within this species. Considering the close genetic resemblance of *Mytilus* spp. and the assumed low gene flow between geographically distant samples (Riginos & Henzler, 2008), simulations were run for a number of predefined *K* values. Based on an initial analysis of *K* up to 18, we found the highest likelihoods for *K* = 3–5. Accordingly, we used this as the basis to identify the major groupings within the species complex. For all simulations, a burn‐in of 10,000 iterations was used followed by 100,000 MCMC repetitions. To evaluate the power of designating individuals as pure or hybrids, we followed the procedure described in Nielsen, Hansen, Ruzzante, Meldrup, and Grønkjær (2003) using the program Hybridlab (Nielsen, Bach, & Kotlicki, 2006). Briefly, we simulated 1,000 individuals of each of the following classes: parentals, F1/F2, and backcrosses. This was done based on the allele frequencies of the reference samples of *M. trossulus*,* M. galloprovincialis*, and *M. edulis* samples identified by initial Structure runs to likely consist exclusively of *M. edulis* individuals (NUS, KOB and WS2). Separate simulations were conducted for *M. edulis* samples from Greenland (NUS, KOB) and the Eastern Atlantic (WS2). The simulated and real individuals were included in a common Structure run (*K* = 4) and 95% confidence intervals for the inferred ancestry of the simulated individuals were recorded and compared to the real individuals.

### 2.6 Population structure of *Mytilus edulis*

A reduced data set was used to assess the population structure in *M. edulis*. Based on the results from the analysis of simulated parentals and hybrids (see results section), we chose to only include individuals with admixture proportions below 0.2, as estimated by Structure. This was done to avoid extensive influence of hybridization on estimates of population divergence, but at the same time allowing for statistical uncertainty regarding whether individuals were pure *M. edulis* individuals or not. No significant differentiation was found between sampled mussels of different size classes from the same location (Tromsø, Nuuk, and Qaanaaq). Consequently, they were pooled prior to downstream analyses of population structure. Pairwise *F*_ST_ estimates were generated with Genepop 4.2, while Structure v2.3.4 was used to estimate the most likely number of genetic clusters. A burn‐in period of 50,000 iterations was chosen followed by 100,000 MCMC repetitions for *K* values 2–4. A hierarchical AMOVA was conducted in Arlequin v.3.5.2.2 (Excoffier & Lischer, 2010) to infer the proportion of genetic variance distributed among the different *M. edulis* clusters and among samples within the clusters detected by Structure (see results section). To visualize the genetically based grouping of *M. edulis* population samples, a multidimensional scaling plot was generated, while a principal component analysis, PCA, was used to illustrate the relationships among *M. edulis* individuals in general and specifically for the Norwegian, Svalbard, and Russian samples to infer the likely origin of Svalbard mussels. The PCA scatter plots were generated in R, using the cmdscale function and the package Adegenet.

### 2.7 Outlier analysis

To identify loci potentially under selection in the “*Mytilus edulis*” data set, the joint distribution of *F*_ST_ and heterozygosity under a hierarchical island model of population structure was examined using Arlequin v.3.5.2.2 (Excoffier & Lischer, 2010) based on the method in Excoffier, Hofer, and Foll (2009). Accounting for the hierarchical population structure reduces the probability of false discoveries (Excoffier et al., 2009). Samples were grouped according to the genetic clustering analyses: (i) Greenlandic samples, (ii) Samples from Norway, the Svalbard archipelago, and Russian waters, and (iii) the Icelandic sample (see the section under Results subsection *Population structure of M. edulis*). The analytical settings for generating 95% and 99% confidence intervals were 20,000 simulations, 100 demes per group, and 10 groups. Loci outside the 95% quantile were considered possible subjects to selection, as these loci deviate more than could be expected under a model of neutral population structure. From this analysis, an exclusive “outlier” data set and a “neutral” data set were created to test the importance of outlier loci for defining the inferred population structure of *M. edulis*; that is, the true connectivity among populations based on neutral processes (drift and migration) could be obscured by loci under divergent selection. For both data sets, overall and pairwise *F*_ST_ estimates were generated in Genepop 4.2, while Structure v2.3.4 with *K* = 2 (using settings as above) was used to investigate whether the population structure found in *M. edulis* based on all loci could be identified from both the “outlier” and “neutral” data sets, or whether they displayed contrasting patterns.

## 3 Results

### 3.1 Summary statistics

Three loci (159069_A, 171383_A, and 170478_A) deviated significantly from HWE in ten samples or more, and they were discarded from further analyses.

In total, 73,206 pairwise tests for LD between loci within samples were performed of which 3,194 tests were significant (4.36%). On average, 154 of 3,485 tests were significant within samples (range 0–986 significant tests). Only three SNP pairs displayed significant LD in more than five samples: 137120_A x BM8E (significant in six samples), 100078_A x 40154_A (significant in eight samples), and 175018_A x 241544_A (significant in 12 samples). Subsequently, one locus from each of the coupled SNP pairs was discarded (BM8E, 241544_A and 40154_A) to eliminate effects of linkage on downstream analyses.

Allelic richness ranged from 1.00 to 1.86 (Table 1), with the lowest values in the Qaanaaq samples (QAS and QAL). The levels of *H*_e_ and *H*_o_ (Table 1) ranged from 0.14 to 0.32 in all samples except for QAS and QAL, which had particularly low values ranging from 0.04 to 0.09. In all samples, *H*_o_ was close to *H*_e_ except for the Upernavik sample (UPE), where *H*_e_ and *H*_o_ was 0.25 and 0.14, respectively, and one of the White Sea samples (WS1) with *H*_e_ and *H*_o_ of 0.32 and 0.24. In the reduced data set, with samples consisting only of inferred *M. edulis,* individuals provided estimates of allelic richness between 1.57 and 1.67 (Appendix 1) and *H*_e_/*H*_o_ values ranging between 0.22 and 0.30.

The overall *F*_ST_ across samples was 0.273. The pairwise *F*_ST_ values ranged between 0 and 0.860 (Table S2) with the highest pairwise *F*_ST_ value between the *M. trossulus* and *M. galloprovincialis* reference samples. Further, high values were found between *M. galloprovincialis* and the N Greenland samples from Qaanaaq; QAS and QAL (0.738 and 0.774) and between *M. trossulus* and all other samples except the three N Greenland samples (QAS, QAL, and UPE).

### 3.2 Identification of hybrids

**Figure 2:**
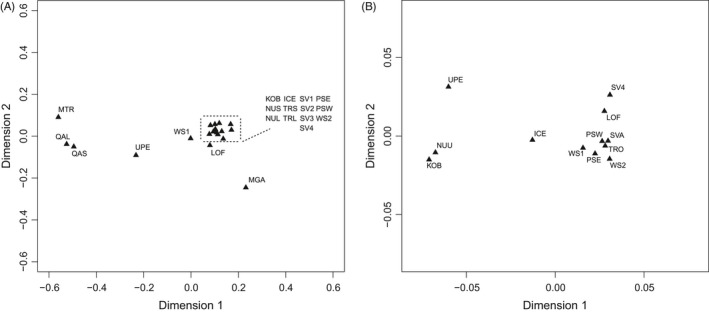
Multidimensional scaling plot of (A) all samples and (B) designated *Mytilus edulis* samples based on pairwise genetic distances among samples. For explanation of sample identification codes, see Table 1. Further codes: NUU comprise of NUS and NUL, TRO comprise of TRS and TRL, and SVA comprise of SV1, SV2, and SV3

**Figure 3:**
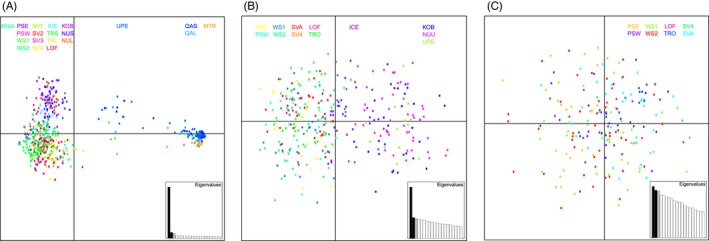
Principal component scatter plot of individual genotypes for (A) all samples (B) *Mytilus edulis* samples, and (C) Norwegian, Svalbard, and Russian samples. For explanation of sample identification codes, see Table 1. Further codes: NUU comprise of NUS and NUL, TRO comprise of TRS and TRL, and SVA comprise of SV1, SV2, and SV3

The multidimensional scaling plot (Fig. 2A) visualizes the genetic differentiation among all samples including the reference samples for *M. trossulus* (MTR) and *M. galloprovincialis* (MGA). The majority of samples clustered together in a “*M. edulis*” cluster. However, the QAS and QAL samples clustered with the *M. trossulus* reference sample, while samples UPE, WS1, and Lofoten (LOF) were located between the three main “species” clusters. UPE and WS1 appeared to be distributed between the *M. trossulus* and *M. edulis* clusters, while LOF was situated between the “*M. edulis*” and “*M. galloprovincialis*” clusters. The clustering of QAS and QAL with MTR, and the inferred separation of UPE, WS1, and LOF from the “*M. edulis*” cluster were further supported by the principal component analysis scatter plot of individual genotypes (Fig. 3A). Most individuals clustered together as a “*M. edulis*” cluster, except for individuals from the UPE and WS1 samples, which appeared to contain individuals distributed between the “*M. trossulus*” and “*M. edulis*” clusters, suggesting that these individuals may be hybrids. The *M. galloprovincialis* reference sample clustered in the proximity of the “*M. edulis*” samples in the multidimensional scaling plot; however, a clear separation between the *M. edulis* and *M. galloprovincialis* samples was still apparent (Fig. 2A).

**Figure 4:**
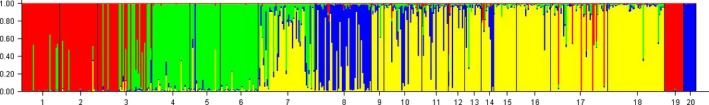
Results of Structure clustering analyses for the full data set for *K* = 4. Samples are 1: QAS, 2: QAL, 3: UPE, 4: NUS, 5: NUL, 6: KOB, 7: ICE, 8: LOF, 9: TRS, 10: TRL, 11: SV1, 12: SV2, 13: SV3, 14: SV4, 15: PSW, 16: PSE, 17: WS1, 18: WS2, 19: MTR, and 20: MGA. For explanation of sample identification codes, see Table 1

The Structure clustering analysis for *K* = 4 (Fig. 4, for *K* = 3 and 5 see Appendix 2) showed that the clusters make biologically sense as they corresponded to *M. trossulus*,* M. galloprovincialis*, Greenlandic *M. edulis*, and other *M. edulis*. This configuration also allowed the identification of *M*. *edulis*/*M. galloprovincialis* or *M. edulis*/*M. trossulus* hybrids. This was supported by the Structure analysis including the simulated parentals and hybrids, which showed relatively narrow 95% confidence intervals for the simulated *M. edulis* parentals regardless of their geographical origin (0.89–0.99 *M. edulis* ancestry for Greenland and 0.92–0.99 for the other *M. edulis*, see Appendix 3). Likewise, the simulated *M. trossulus*,* M. galloprovincialis* parentals suggested very high power for identifying *M*. *edulis*/*M. galloprovincialis* or *M. edulis*/*M. trossulus* hybrids. However, as only two relatively small samples of *M. trossulus* and *M. galloprovincialis* provided the foundation for the simulations, we conservatively chose an admixture level of 20% as the cutoff point between *Mytilus* spp. parentals and *M*. *edulis*/*M. galloprovincialis* or *M. edulis*/*M. trossulus* hybrids. This was done in order to allow for uncertainty caused by population structure and missing genotypes within the samples of real individuals. This approach enabled the construction of an exclusive “*Mytilus edulis*” data set. When using *K* = 3 the analysis was unable to split the samples into the three a priori defined species groups (Appendix 2B).

The inferred proportion (using the 20% criterion) of the different *Mytilus* species and hybrids in each of the geographical samples (Fig. 1) show that *M. edulis* is the most common species within the sampled subarctic and Arctic populations, where pure *M. edulis* specimens constitute approximately 66% of all sampled individuals. Pure *M. edulis* were present in all samples except for QAS and QAL, which were mainly *M. trossulus* (87%–90%), with few individuals (3–4) showing evidence of *M. edulis* hybridization. Only two samples, UPE and WS1, contained both pure *M. edulis* and *M. trossulus* individuals. The UPE sample contained approximately 51% *M. trossulus* and 33% *M. edulis* and 14% *M. edulis*/*M. trossulus* hybrids, while the WS1 sample was comprised of 9% *M. trossulus*, 80% *M. edulis*, 9% *M. edulis*/*M. trossulus* hybrids, and 2% *M. edulis*/*M. galloprovincialis* hybrids. The distribution of *M. galloprovincialis* individuals is mainly restricted to samples from the Norwegian coast and the Svalbard archipelago (34 and 4 individuals, respectively). A single apparent *M. galloprovincialis* individual was found in the sample of large mussels from Nuuk (NUL). The LOF sample contained the highest number of *M. galloprovincialis* observed—64% and further 11% *M. edulis* and 22% *M. edulis*/*M. galloprovincialis* hybrids. In cases where more than a few hybrids were found, the distribution of admixture estimates of real individuals was compared to the simulated hybrids. In all cases, different classes of hybrids (F1, F2, and backcrosses) were suggested. However, as explained above the comparison of real and simulated individuals should be interpreted with caution.

### 3.3 Population structure of *Mytilus edulis*

The overall *F*_ST_ for all samples identified as *M. edulis* was 0.048. Pairwise *F*_ST_ values ranged from 0 to 0.113 with the highest values between the Greenlandic samples and the Norwegian, Svalbard, and Russian samples (Table S3). The lowest *F*_ST_ values were found between geographically proximate samples such as the two White Sea samples (WS1 and WS2) and the two sampling sites in Svalbard (SVA and SV4). For sites with samples of different size classes, *F*_ST_ estimates ranged between 0.001 for the Nuuk samples (NUS and NUL) and 0.018 for the Tromsø samples (TRS and TRL) (Supplementary Table S3). The low *F*_ST_ for Nuuk samples indicates short‐term temporal stability of genetic population structure. The higher *F*_ST_ estimate for Tromsø mussels was not significant, thus allowing the pooling of size classes for the downstream analyses.

**Figure 5:**
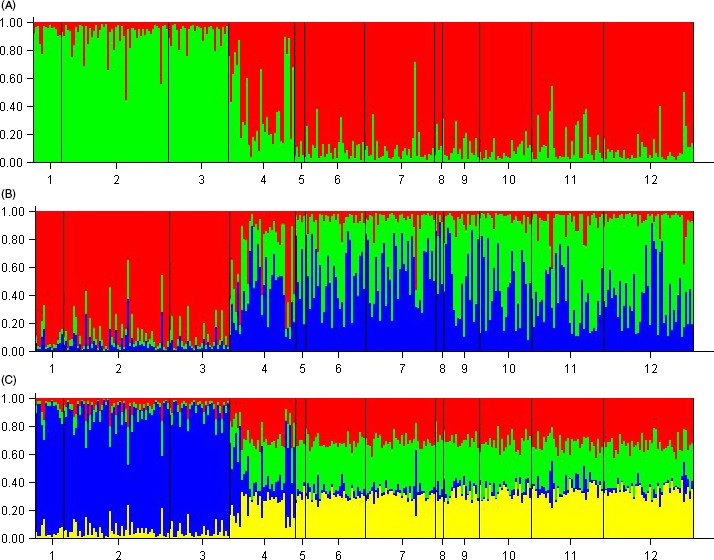
Results from clustering analyses of the “*Mytilus edulis*” data set with: (A) *K* = 2, (B) *K* = 3, and (C) *K* = 4. Samples are as follows: 1: UPE, 2: NUU comprising of NUS and NUL, 3: KOB, 4: ICE, 5: LOF, 6: TRO comprising of TRS and TRL, 7: SVA comprising of SV1, SV2, and SV3, 8: SV4, 9: PSW, 10: PSE, 11: WS1, and 12: WS2. For explanation of sample identification codes, see Table 1

The cluster analysis of the “*Mytilus edulis*” data set (*K* = 2–4) showed a clear clustering of samples, essentially separating the Greenlandic samples from the other samples (Fig. 5). The likelihood of *K* = 2 was highest splitting the *M. edulis* samples into two groups; the Greenlandic samples versus the Norwegian, Svalbard, and Russian samples and identifying the Icelandic sample a mixture of eastern and western Atlantic gene pools (Fig. 5A). The plots for *K* = 3 and *K* = 4 added no additional biologically sensible information.

The hierarchical AMOVA for the three groups (Greenlandic, Icelandic, and Norwegian/Svalbard/Russian) provided an estimated variance of 5.78% among groups and 0.43% among samples within groups. The multidimensional scaling plot of population samples (Fig. 2B) and the principal component analysis scatter plot of individual genotypes (Fig. 3B) further supported the population structure of *M. edulis* inferred by Structure with three groups: (i) Greenlandic samples, (ii) Norwegian, Svalbard, and Russian samples, and (iii) the Icelandic sample found between the two main clusters inferred by axis 1.

The principal component analysis scatter plot including only Norwegian, Svalbard, and Russian samples (Fig. 3C) did not provide a clear separation of individuals as these individuals were scattered with no apparent pattern.

### 3.4 Outlier analysis

**Figure 6:**
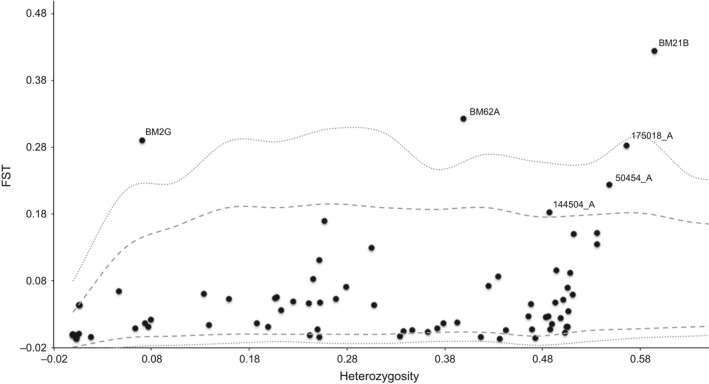
*F*_ST_ outlier analyses in Arlequin v3.5.1.3 utilizing the hierarchical island model. *Black solid dots* denote loci, and *gray dashed* and *dotted lines* indicate 95% and 99% confidence intervals, respectively. Loci outside the 95% and 99% confidence intervals are suggested to be under selection

The outlier tests identified six loci as *F*_ST_ outliers, with six loci significant at the 5% level and three at the 1% level. All of these outliers are high *F*_ST_ outliers (Fig. 6) indicating diversifying selection (Beaumont & Nichols, 1996), although a few of them could represent the upper tail of the neutral *F*_ST_ distribution. Also, a strong genetic cline as observed here is known to sometimes overestimate the number of loci under diversifying selection (Strand, Williams, Oleksiak, & Sotka, 2012). Furthermore, introgression between *Mytilus* spp. has been found to cause high *F*_ST_ outliers (Gosset and Bierne 2012). Pairwise *F*_ST_ values ranged from 0 to 0.059 for the “neutral” data set and from 0 to 0.474 for the “outlier” data set (Tables S4 and S5). The Structure analyses for both the “neutral” and “outlier” data set also supported the initial population structure separating Greenlandic samples from the Norwegian, Svalbard, and Russian samples and with the Icelandic sample of admixed origin (Appendices 4 and 5).

## 4 Discussion

### 4.1 Distribution of *Mytilus* spp. in the Arctic

Baseline information of species distribution and their genetic composition is imperative in order to quantify the impacts of climate change on species distribution ranges, biodiversity, and the effects of hybridization between species and populations (Gardner, Zbawicka, Westfall, & Wenne, 2016). Molecular genetic knowledge is a key measure to identify the distribution of invasive congener species (Geller, Darling, & Carlton, 2010), which may cause cascading ecosystem effects. Despite congener species appearing morphologically similar, interspecific variation in ecology and physiology may impact population fitness (Fly & Hilbish, 2013; Fraïsse et al., 2016; Somero, 2005). In the Arctic, baselines studies on genetic variation and species abundance are largely absent but urgently needed (Bluhm et al., 2011; Wassmann, Duarte, Agusti, & Sejr, 2011). Pioneer work should therefore focus on keystone model species (such as *Mytilus*), because of their disproportionally large effect on their environment.

*Mytilus* spp. were found pan‐Arctic (although only one individual of *M. galloprovincialis* was identified in Greenland). Generally, *M. edulis* was the most common species making up approximately 66% of all sampled individuals. The biogeographic structures of the three *Mytilus* spp. reflect the major current systems of the region. Pure populations were mainly found in regions (such as W Greenland and the Pechora Sea) with a lower influence of Pacific and Atlantic water, than other sampling sites. Northwards currents from boreal waters facilitate larvae dispersal from southern populations (Berge et al., 2005; Renaud, Sejr, Bluhm, Sirenko, & Ellingsen, 2015). For instance, the northward flowing current regimes (such as the Norwegian Current) allows non‐Arctic species to extend their range into the Arctic from the Atlantic or Pacific Ocean (Bluhm et al., 2011; Fetzer & Arntz, 2008; Sirenko & Gagaev, 2007). Ocean currents also explain why *Mytilus* spp. remain absent in NE Greenland despite the environmental resemblance of NW Greenland with regard to temperatures and ice conditions (Sejr, Blicher, & Rysgaard, 2009). In general, the NE Greenland shelf is considered biogeographically different from the rest of Greenland (Piepenburg et al., 2011). The absence of *Mytilus* mussels in NE Greenland is likely a result of dispersal barriers due to the lack of an downstream source population, as the East Greenland Current flows from north to south, exemplifying how outflow shelves may respond slowly to climatic changes (Renaud et al., 2015). This is further supported by the presence of *Mytilus* mussels in SE Greenland, at Tasiilaq (Ammassalik, 65°N) (Ockelmann, 1958), which is influenced by a branch of the Irminger Current from the Atlantic Ocean.

The present study highlights the need for further genetic studies in the region as a *M. trossulus* population was found in the most northern sampled region of NW Greenland (77°N) with *M. edulis* populations residing in SW Greenland (64°N). This discovery was unexpected, as a seemingly established *M. trossulus* population has not been found in the high Arctic prior to this study. Several possible mechanisms could explain the presence of *M. trossulus* in Qaanaaq and Upernavik. First, these populations could have survived in a refugium near NW Greenland during the last glacial period. Glacial refugia are known from North Atlantic temperate regions and evidence suggests that *M. edulis* may have survived north of the ice margin (Maggs et al., 2008; Riginos & Henzler, 2008). Second, there could be a contemporary spread of *M. trossulus* from the Pacific Ocean. Jones et al. (2003) found that waters around NW Greenland contained high levels of phosphate indicating Pacific water being transported into this area. Also, there are a few reports of live *M. trossulus* in Arctic Alaska and Canada (Feder et al., 2003), so the spread of planktonic larvae from the Canadian Arctic could be possible. A third scenario could be that *M. trossulus* spread to Arctic Greenland from the East coast of Canada. However, as the West Greenland Current moves along the coast from south to north, and *Mytilus* mussels are expected to disperse with rather than against currents, this scenario seems unlikely (McQuaid & Phillips, 2000). Finally, *Mytilus* spp. are known to disperse by human activities and can survive long distances and fluctuating temperatures (Lee & Chown, 2007). Qaanaaq is situated less than 150 km from the US Thule Air Base, which receives supplies by US ships; this is providing an alternative dispersal route of *M. trossulus* from the north Pacific.

The invasive *M. galloprovincialis* appeared widespread from Greenland to the Pechora Sea. In Norway, *M. galloprovincialis* appears common along the coastline (Brooks & Farmen, 2013), and the discovery of *M. galloprovincialis* in Svalbard suggests colonization by ocean currents as hypothesized by Berge et al. (2005) or ship traffic from the Norwegian mainland (Ware et al., 2014).

### 4.2 *Mytilus* hybrid zones in the Arctic

Most sampling locations displayed varying degrees of hybridization and introgression between different *Mytilus* spp. and only four locations contained apparently pure populations (Fig. 1). Introgression can affect a population's fitness and vulnerability to climate change. In the study region, hybrid zones were found in Norway, Svalbard, and Greenland, with the highest abundance of the invasive *M. galloprovincialis* found along the Norwegian coast, especially in Lofoten (68°N) further supporting the findings by Brooks and Farmen (2013) and Riginos and Henzler (2008). Additionally, a surprisingly high amount of *M. galloprovincialis* was found at Svalbard. We also found evidence of limited introgression of *M. galloprovincialis* in the Russian and Icelandic samples, and the ecological consequences of invasive mussels in these regions need to be studied further. In the White Sea, *M. trossulus* individuals were only recorded in one of two locations. This small‐scale regional variation in species composition was also observed by Väinölä and Strelkov (2011), who also found *M. trossulus* and *M. edulis*/*M. trossulus* hybrids but to a much lesser extent than *M. edulis*. It is believed that the expansion of *M. trossulus* in the White Sea is most likely facilitated by ships (Väinölä & Strelkov, 2011). This explains the fact that populations dominated by *M. trossulus* are confined to sites with harbors and seaports, while *M. edulis* inhabit all the coastline of the White Sea, where the substrates are appropriate. In the present study, the sample WS1 that contained *M. trossulus* and their hybrids were collected directly in the area of the White Sea Biological Station Kartesh, which has a regular ship connection with Chupa, a small town in Kandalaksha Bay. Recently, *M. trossulus* was found in the Chupa harbor (Katolikova, Khaitov, Vänölä, Gantsevich, & Strelkov, 2016), where ship traffic from the Barents Sea has been relatively intensive. In contrast, the WS2 site with pure *M. edulis* in the sample is located on an uninhabited island Kondostrov in the Onega Bay, which is far from the towns with intensive ship traffic.

### 4.3 Population structure of *Mytilus edulis*

The genetic structure of the *M. edulis* populations in this study revealed a significant split between *M. edulis* samples from each side of the Atlantic, with Icelandic *M. edulis* appearing as an admixture of the two gene pools. This divergence of W and E Atlantic populations is in line with the findings of Riginos and Henzler (2008) and Waltari and Hickerson (2013), who suggested that *M. edulis* survived in a W Atlantic glacial refugium. Furthermore, Riginos et al. (2004) found low gene flow across the Atlantic, providing an explanation for the continuing divergence of *M. edulis* populations from W and E Atlantic coasts. These studies primarily looked at mitochondrial DNA, but their results are strongly supported by the SNP analysis presented here. This, however, contrasts to the meta‐population analysis of polychaete and echinoderm populations in the Arctic showing high gene flow between populations (Hardy et al., 2011). This difference in gene flow patterns between different species with long planktonic larval stage further highlights the necessity of understanding the population structure within species to best conserve biodiversity in the Arctic.

In general, *F*_ST_ values between samples from Norway, Svalbard, and Russia and the Icelandic sample are lower than between the Icelandic sample and Greenlandic samples. Śmietanka, Burzyński, Hummel, and Wenne (2014) suggested a single glacial Atlantic refugium founding European *M. edulis*. However, our studied sample from Iceland suggests the population to consist of individuals of mixed ancestry. Further analyses of their origin/history could be elucidated by conducting additional analysis of samples from both sides of the Atlantic. Considering that the major North Atlantic Current reaches Iceland from the east, it is perhaps more likely that Iceland would be recruiting spat from East Atlantic populations. This is also inferred by Riginos and Henzler (2009), who found postcolonization gene flow from northern Europe to Iceland.

The outlier tests identified six loci as *F*_ST_ outliers at the 5% significance levels. All of these outliers are high *F*_ST_ outliers (Fig. 6) indicating diversifying selection (Beaumont & Nichols, 1996). However, a strong genetic cline as observed here is known to sometimes overestimate the number of loci under diversifying selection (Strand et al., 2012). Furthermore, introgression between *Mytilus* spp. have been found to cause high *F*_ST_ outliers (Gosset & Bierne, 2012), and this result should be interpreted with some caution. Still, we find that the pattern of population structure is the same for the “neutral” and the “outlier” data sets (Appendices 4 and 5), suggesting that patterns of neutral population structure is correlated with adaptive evolution in response to divergent local environmental conditions. Temperature influences the large‐scale geographical distribution of species (Sunday, Bates, & Dulvy, 2011); however, on a local scale other factors including predation, the presence of sea ice, suitable habitats, water current, and salinity can influence the distribution of intertidal species (Høgslund, Sejr, Wiktor, Blicher, & Wegeberg, 2014; Kroeker et al., 2016; Paine, 1974), and these conditions are very different between W Greenland and the other sampling sites (Rayner et al., 2003). Still, the high divergence between samples from the Eastern Atlantic and Greenland cannot be explained alone by loci subject to selection. *F*_ST_ values for the “neutral” data set are still high (Table S4) suggesting a high degree of isolation between groups. This isolation in turn may have facilitated local adaptation at this rather large geographical scale. For more specific insights on the environmental factors responsible for local adaptation, the geographical scale, and its genomewide significance, a more elaborate sampling design is warranted including more regional samples and a higher degree of genomic coverage.

### 4.4 Implications for conservation of marine species in the face of climate change

The effects of global warming increase the spread and associated threat of nonindigenous species across the globe (Gardner et al., 2016; Hellmann, Byers, Bierwagen, & Dukes, 2008; Saarman & Pogson, 2015). A study by Wisz et al. (2015) predicted that continued warming of the Arctic could open the Bering Strait and thus facilitate a Pacific–Arctic exchange of nonindigenous species, which could have adverse impact on Arctic biodiversity. Moreover, human activities are short‐cutting natural dispersal barriers for nonindigenous species (Carlton & Geller, 1993), posing a global risk of spreading these to novel regions. In this regard, especially ship traffic facilitates dispersal (e.g. in ballast water and hull fouling; Chan, MacIsaac, & Bailey, 2015; Geller et al., 1994; Ware et al., 2014). Such intrusions of nonindigenous species into the Arctic have already occurred (e.g. Pacific king crabs *Paralithodes camtschaticus* and bluefin tuna *Thunnus thynnus*; CAFF 2013; MacKenzie, Payne, Boje, Hoyer, & Siegstad, 2014; Oug, Cochrane, Sundet, Norling, & Nilsson, 2011), and Saarman and Pogson (2015) found that the nonindigenous *M. galloprovincialis* pose an ecological threat to *M. trossulus* along the Californian coast as it had displaced and continues to displace the native *M. trossulus*. The surprisingly broad distribution of *M. galloprovincialis* in the Arctic therefore highlights the benefit of using genetic tools and stresses the need for developing measures to detect and identify nonindigenous species and pathways of introduction, to understand and reduce the threat of invasive species in the Arctic.

Prior to the current investigation, multiple studies have assumed *Mytilus* mussels in the Arctic to be exclusively *M. edulis* (Berge et al., 2005; Hansen, Hanken, Nielsen, Nielsen, & Thomsen, 2011; Jensen, 1905; Strand & Asmund, 2003). The identification of three *Mytilus* spp. across the Arctic has implications for ecological and ecotoxicological research in the region. *Mytilus* mussels are extensively used in biological monitoring programs (Wenne et al., 2016). However, interspecific differences in physiology and responses to environmental pollutants have been reported (Brooks, Farmen, Heier, Blanco‐Rayon, & Izagirre, 2015; Fly & Hilbish, 2013), and thus, the lack of genetic knowledge could seriously affect the conclusions of ongoing biological monitoring. We therefore emphasize the importance of applying genetic tools to document species status, when conducting ecological, ecotoxicological, and physiological studies.

Moreover, assuming that the distribution and genetic connectivity between regions observed in this study is to be a first approximation representative for benthic invertebrates in general, several important observations were made related to quantifying changes in species distribution in a warmer Arctic. A number of congener species exists, which display different responses to changes in temperature. The genetic connectivity and inferred gene flow are closely linked to major ocean currents, which means that predicting range changes purely based on future climate predictions without considering dispersal potential or barriers can be misleading. In fact, changes in ocean currents and thereby in supply of potential colonizers may be a more important driver of change than warming per se. This has previously been demonstrated by the species changes observed during the large northward expansion of Atlantic water in the Barents Sea and along the W Greenland coast in the 1930s (Drinkwater, 2006). Genetically isolated areas like outflow shelves without upstream source populations (such as, NE Greenland) appear to be especially vulnerable to human vectors (such as shipping) as the absence of several species here likely reflects lacking postglacial invasion rather than adverse climatic conditions. Finally, NW Greenland *M. trossulus* populations with an affinity to the Pacific suggest that exchange of species from the Pacific across the Arctic and into the Atlantic is taking place. However, all of these factors should be further validated through urgently needed studies documenting current distribution and genetic composition of marine species in the Arctic.

## Data Archiving Statement

Data available from the Dryad Digital Repository: http://dx.doi.org/10.5061/dryad.438h3.

## Conflict of Interest

The authors declare no conflict of interest.

## Appendix 1 *H*_e_, *H*_o_ and allelic richness for the “*Mytilus edulis*” data set.

### 1.1

Estimates of expected (*H*_e_) and observed (*H*_o_) heterozygosities and the allelic richness for the samples only including *M. edulis*.

**Table:**

Code	*H*_e_	*H*_o_	Allelic richness
UPE	00.24	00.24	1.54
NUS	00.25	00.23	1.53
NUL	00.23	00.22	1.54
KOB	00.25	00.22	1.54
ICE	00.28	00.27	1.60
LOF	00.26	00.28	1.58
TRS	00.27	00.29	1.64
TRL	00.28	00.24	1.50
SV1	00.27	00.25	1.59
SV2	00.27	00.27	1.62
SV3	00.26	00.25	1.58
SV4	00.27	00.25	1.60
PSW	00.30	00.29	1.66
PSE	00.30	00.29	1.67
WS1	00.28	00.25	1.62
WS2	00.29	00.27	1.66

## Appendix 2 Structure analysis for the full data set.

### 2.1

Results of Structure clustering analyses for the full data set with: (a) *K* = 3 and (b) *K* = 5. Samples are 1: QAS, 2: QAL, 3: UPE, 4: NUS, 5: NUL, 6: KOB, 7: ICE, 8: LOF, 9: TRS, 10: TRL, 11: SV1, 12: SV2, 13: SV3, 14: SV4, 15: PSW, 16: PSE, 17: WS1, 18: WS2, 19: MTR, and 20: MGA. For explanation of sample identification codes, see Table 1.

## Appendix 3 Confidence intervals

### 3.1

95% confidence intervals for the inferred ancestry for simulated parentals and hybrids analyzed with Structure (*K* = 4). Ancestry estimates for the two inferred *Mytilus edulis* clusters (Greenland and Eastern Atlantic) are pooled. See text for explanation.

**Table:**

Simulated individuals	*Mytilus edulis*	*Mytilus trossulus*	*Mytilus galloprovincialis*
Greenland			
*M. edulis*	0.89–0.99	0.00–0.02	0.01–0.10
*M. trossulus*	0.00–0.01	0.99–1.00	0.00–0.01
*M. edulis/M. trossulus F1*	0.34–0.54	0.44–0.56	0.01–0.10
*M. edulis/M. trossulus F2*	0.36–0.59	0.39–0.60	0.01–0.09
*M. edulis/M. trossulus backcross M. edulis*	0.60–0.82	0.15–0.34	0.01–0.10
*M. edulis/M. trossulus backcross M. trossulus*	0.14–0.31	0.67–0.82	0.01–0.07
Eastern Atlantic			
*M. edulis*	0.92–0.99	0.00–0.02	0.01–0.07
*M. trossulus*	0.00–0.01	0.99–1.00	0.00–0.01
*M. galloprovincialis*	0.01–0.02	0.00–0.01	0.97–0.99
*M. edulis/M. trossulus F1*	0.43–0.55	0.43–0.54	0.01–0.06
*M. edulis/M. trossulus F2*	0.38–0.60	0.37–0.59	0–01–0.09
*M. edulis/M. trossulus backcross M. edulis*	0.70–0.76	0.20–0.25	0.04–0.05
*M. edulis/M. trossulus backcross M. trossulus*	0.17–0.33	0.65–0.80	0.01–0.08
*M. edulis/M. galloprovincialis F1*	0.37–0.68	0.00–0.02	0.31–0.62
*M. edulis/M. galloprovincialis F2*	0.32–0.73	0.00–0.03	0.26–0.67
*M. edulis/M. galloprovincialis backcross M. edulis*	0.61–0.96	0.00–0.02	0.03–0.39
*M. edulis/M. galloprovincialis backcross M. galloprovincialis*	0.14–0.39	0.00–0.02	0.60–0.86

## Appendix 4 Structure analysis for the “*Mytilus edulis*” “neutral” data set.

### 4.1

Results from clustering analysis (*K* = 2) for the “*Mytilus edulis*” data set only including SNPs not documented as outliers. Samples are 1: UPE, 2: NUU comprising of NUS and NUL, 3: KOB, 4: ICE, 5: LOF, 6: TRO comprising of TRS and TRL), 7: SVA comprising of SV1, SV2 and SV3, 8: SV4, 9: PSW, 10: PSE, 11: WS1, and 12: WS2. For explanation of sample identification codes, see Table 1.

## Appendix 5 Structure analysis for the “*Mytilus edulis*” “outlier” data set.

### 5.1

Results from clustering analysis (*K* = 2) for the “*Mytilus edulis*” data set only including the six outlier SNPs Samples are 1: UPE, 2: NUU comprising of NUS and NUL, 3: KOB, 4: ICE, 5: LOF, 6: TRO comprising of TRS and TRL), 7: SVA comprising of SV1, SV2, and SV3, 8: SV4, 9: PSW, 10: PSE, 11: WS1, and 12: WS2. For explanation of sample identification codes, see Table 1.
