# Supplementary material for: Genetic diversity and connectivity within Mytilus spp. in the subarctic and Arctic
Source: Evol Appl. 2016 Oct 26;10(1):39–55. doi: 10.1111/eva.12415 (PMC5192891; doi:10.1111/eva.12415)
Supplement: Supplementary file 1 [file EVA-10-39-s001.docx]

**Supplementary tables**

**Table S1**. SNP properties including location (if known), GenBank annotation and the genotyped alleles observed in the three different *Mytilus* species. The 96 SNPs consist of 19 SNPs from a previous publication on *Mytilus* spp. genetic structure (Zbawicka et al. 2012; Zbawicka et al. 2014) and 77 SNPs from RAD sequencing of genomic *M. edulis* DNA at the University of Stirling.

| SNP | Location | GenBank annotation | Allele | *M. edulis* | *M. trossulus* | *M. galloprovincialis* | |  |
| --- | --- | --- | --- | --- | --- | --- | --- | --- |
| BM1C | CSD | KJ871031 | A/T | A/T, T/T, A/A | T/T | A/T, A/A | |  |
| BM11A | Ribosomal protein L22 | KJ871041 | A/G | A/G, A/A | G/G | A/A | |  |
| BM12A | Ribosomal protein L23a | KJ871042 | C/T | C/T, C/C, T/T | T/T (C/C) | C/C | |  |
| BM202A* | H3 histone gene | AY267749.1 | A/C | NA | NA | NA | |  |
| BM203D | H2A histone gene | AY267757.1 | A/T | A/A, A/T | T/T | A/A | |  |
| BM21B | qm-like protein | KJ871047 | C/G | C/G, G/G, C/C | C/C | G/G | |  |
| BM26B | UnKnown13 | KJ871050 | A/T | A/T, T/T, A/A | A/A, A/T | T/T | |  |
| BM2G | UnKnown05 | KJ871032 | G/T | G/G, T/T (G/T) | T/T | G/G | |  |
| BM35E | Ribosomal protein L7 | KJ871055 | C/T | C/T, C/C, T/T | T/T | C/C | |  |
| BM54A | ETC_C1_NDUFA4 | KJ871060 | A/G | A/A | G/G | A/A | |  |
| BM62A | Ribosomal L13e | KJ871064 | A/G | A/G, A/A, G/G | NA | NA | |  |
| BM64A | Ribosomal protein L35 | KJ871065 | C/T | C/C | T/T | C/C | |  |
| BM76B | UnKnown01 | KJ871068 | A/G | A/A | G/G | A/A, A/G | |  |
| BM79B | DUF1143 | KJ871070 | A/G | A/G, A/A | G/G | A/A | |  |
| BM8E* | Ribosomal protein L3 | KJ871038 | A/G | A/G, A/A, G/G | A/A | G/G | |  |
| BM92B | UnKnown06 | KJ871074 | A/T | A/T, T/T, A/A | T/T | A/A | |  |
| BM93B | Ribosomal protein L27e | KJ871075 | C/T | C/T, T/T | C/C | T/T | |  |
| BM96A | UnKnown09 | KJ871076 | A/G | G/G | A/A | G/G | |  |
| BM96B | UnKnown09 | KJ871076 | C/T | C/T, T/T | C/C | T/T | |  |
| 100078_A | - | - | A/T | A/T, A/A, T/T | T/T | T/T | |  |
| 10532_A | - | - | C/T | C/T, C/C, T/T | C/C | C/C | |  |
| 109186_A | - | - | A/G | A/G, A/A, G/G | NA (A/A) | A/A | |  |
| 110911_A* | - | - | A/C | A/C, A/A, C/C | C/C | C/C | |  |
| 113822_A | - | - | A/T | A/T, T/T, A/A | T/T | T/T | |  |
| 122961_A* | - | - | A/G | A/G, A/A, G/G | A/A | A/A | |  |
| 130289_A | - | - | A/G | A/G, A/A, G/G | A/A (A/G) | A/G, A/A | |  |
| 137120_A | - | - | A/T | A/T, A/A, T/T | T/T | A/T | |  |
| 137515_A | - | - | G/T | G/T, G/G, T/T | G/G | G/T, T/T | |  |
| 140245_A | - | - | C/G | C/G, G/G, C/C | G/G | G/G | |  |
| 144504_A | - | - | G/T | G/T, G/G, T/T | NA | G/G | |  |
| 151552_A | - | - | C/T | C/T, C/C, T/T | C/C | C/T, C/C | |  |
| 155702_A | - | - | A/T | A/G, A/A, G/G | A/A | A/A | |  |
| 156571_A | - | - | A/G | A/G, A/A, G/G | G/G | A/G, G/G | |  |
| 158627_A | - | - | G/T | G/T, G/G, T/T | T/T | G/T, T/T | |  |
| 158935_A | - | - | C/T | C/T, C/C, T/T | C/C | C/T, C/C | |  |
| 159069_A* | - | - | A/C | A/C, A/A, C/C | C/C | A/C, A/A, C/C | |  |
| 162610_A | - | - | A/T | A/T, T/T, A/A | T/T (A/T) | T/T | |  |
| 164355_A | - | - | A/T | A/T, T/T, A/A | A/A | NA | |  |
| 170118_A | - | - | C/T | C/T, C/C, T/T | T/T | T/T | |  |
| 170478_A* | - | - | A/G | A/G, A/A, G/G | NA | A/G, G/G | |  |
| 17122_A | - | - | C/T | C/T, C/C, T/T | T/T | T/T | |  |
| 171383_A* | - | - | A/G | A/A, G/G | G/G | G/G | |  |
| 174302_A | - | - | A/T | A/T, A/A, T/T | NA | T/T | |  |
| 175018_A | - | - | A/G | A/G, A/A, G/G | A/A | A/A, A/G | |  |
| 176084_A | - | - | C/T | C/T, C/C, T/T | T/T, NA | T/T | |  |
| 184182_A | - | - | A/G | A/G, A/A, G/G | G/G | A/G, G/G | |  |
| 189337_A | - | - | C/T | C/T, C/C, T/T | T/T | C/T, T/T | |  |
| 195676_A | - | - | A/T | A/T, T/T, A/A | T/T | T/T | |  |
| 197432_A | - | - | A/T | A/T, T/T, A/A | T/T | T/T | |  |
| 200203_A | - | - | C/T | C/T, C/C, T/T | C/C | C/C | |  |
| 200448_A* | - | - | C/T | C/T, C/C, T/T, NA | NA | | NA | |
| 210133_A | - | - | A/C | A/C, A/A | NA | | A/A (C/C) | |
| 210137_A | - | - | A/T | A/T, T/T, A/A | A/T, A/A, T/T | | A/T, A/A | |
| 21391_A | - | - | A/C | A/C, A/A, C/C | C/C | | C/C | |
| 216174_A | - | - | C/T | C/T, C/C, T/T | C/C | | C/C | |
| 222830_A | - | - | G/T | G/T, G/G, T/T | G/G | T/T | |  |
| 22536_A | - | - | C/G | C/G, G/G | G/G | C/G, G/G | |  |
| 228380_A | - | - | A/T | A/A | A/A | A/A | |  |
| 231634_A* | - | - | A/G | NA | NA | NA | |  |
| 234828_A | - | - | A/C | A/C, A/A, C/C | C/C | C/C | |  |
| 235557_A | - | - | A/C | A/C, A/A, C/C | NA, A/A | A/C, A/A | |  |
| 238086_A | - | - | A/T | A/T, T/T, A/A | T/T | T/T | |  |
| 241544_A* | - | - | C/T | C/T, C/C, T/T | T/T | T/T | |  |
| 26142_A* | - | - | A/C | A/C, A/A | NA, A/A | A/C, A/A, NA | |  |
| 26775_A* | - | - | A/T | A/T, T/T, A/A | NA (A/A) | T/T | |  |
| 269403_A | - | - | A/G | G/G | G/G | G/G | |  |
| 29987_A | - | - | C/T | C/T, C/C, T/T | NA | T/T (C/C) | |  |
| 31051_A | - | - | C/T | C/T, C/C, T/T | C/T, C/C | T/T | |  |
| 31305_A | - | - | C/T | C/T, C/C, T/T | C/C | C/T, C/C | |  |
| 317253_A | - | - | G/T | G/T, G/G, T/T | G/G | G/G | |  |
| 33794_A | - | - | A/G | A/G, A/A, G/G | G/G | G/G | |  |
| 33844_A | - | - | G/T | G/G | G/G | G/G | |  |
| 346399_A | - | - | A/G | A/G, A/A, G/G | G/G | G/G, A/G | |  |
| 357886_A | - | - | A/G | A/G, A/A, G/G | A/A | A/A | |  |
| 35890_A | - | - | C/T | C/T, C/C, T/T | C/C, T/T | C/T, C/C | |  |
| 40154_A* | - | - | A/C | A/C, A/A, C/C | NA, A/A | A/A | |  |
| 40763_A | - | - | A/G | A/G, A/A, G/G | G/G | G/G | |  |
| 408293_A | - | - | C/T | C/T, C/C, T/T, NA | NA | NA | |  |
| 43333_A | - | - | A/G | A/G, A/A, G/G | G/G | A/G, A/A, G/G | |  |
| 437439_A | - | - | C/T | C/T, C/C | C/C | C/T, C/C | |  |
| 44073_A* | - | - | A/C | A/C, A/A, C/C | NA (A/A) | A/C, A/A | |  |
| 45739_A | - | - | G/T | G/T, G/G, T/T | T/T | NA | |  |
| 467930_A | - | - | C/T | C/T, C/C, T/T | T/T | C/T, T/T | |  |
| 49216_A | - | - | A/C | A/C, A/A, C/C | A/A | A/A | |  |
| 50454_A | - | - | C/T | C/T, C/C, T/T | C/C | C/T, T/T | |  |
| 59317_A | - | - | A/G | A/G, A/A, G/G | A/A | A/A | |  |
| 62480_A | - | - | A/C | A/C, A/A, C/C | C/C | A/C, C/C | |  |
| 6389_A | - | - | A/G | A/G, A/A | A/G, A/A | A/A | |  |
| 67577_A | - | - | A/C | A/C, A/A, C/C | NA, A/A | A/A | |  |
| 72530_A | - | - | G/T | G/T, G/G, T/T | G/G | G/G | |  |
| 74526_A | - | - | C/G | C/G, G/G, C/C | NA | C/G, C/C, G/G | |  |
| 80460_A | - | - | A/T | A/T, T/T, A/A | T/T | T/T (A/T) | |  |
| 86520_A* | - | - | A/T | T/T | T/T | T/T | |  |
| 9749_A | - | - | C/T | C/T, C/C | C/C | C/C | |  |
| 98199_A | - | - | A/C | A/C, A/A, C/C | A/A | A/C, C/C | |  |
| 98787_A | - | - | C/G | C/G, G/G, C/C | NA | C/C | |  |

* indicates SNPs removed prior to analyses.

(): Genotypes in brackets were only observed once within the species.

**Table S2**. Pairwise F_ST_ values among all samples.

| **F_ST_**^a^ | QAS^b^ | QAL | UPE | NUS | NUL | KOB | ICE | LOF | TRS | TRL | SV1 | SV2 | SV3 | SV4 | PSW | PSE | WS1 | WS2 | MTR |
| --- | --- | --- | --- | --- | --- | --- | --- | --- | --- | --- | --- | --- | --- | --- | --- | --- | --- | --- | --- |
| QAL | 0.0000 |  |  |  |  |  |  |  |  |  |  |  |  |  |  |  |  |  |  |
| UPE | 0.1464 | 0.1788 |  |  |  |  |  |  |  |  |  |  |  |  |  |  |  |  |  |
| NUS | 0.5962 | 0.6247 | 0.2860 |  |  |  |  |  |  |  |  |  |  |  |  |  |  |  |  |
| NUL | 0.6027 | 0.6328 | 0.2811 | 0.0008 |  |  |  |  |  |  |  |  |  |  |  |  |  |  |  |
| KOB | 0.6067 | 0.6351 | 0.2970 | 0.0000 | 0.0011 |  |  |  |  |  |  |  |  |  |  |  |  |  |  |
| ICE | 0.5731 | 0.5958 | 0.3052 | 0.0513 | 0.0453 | 0.0516 |  |  |  |  |  |  |  |  |  |  |  |  |  |
| LOF | 0.5759 | 0.5954 | 0.3202 | 0.1298 | 0.1165 | 0.1369 | 0.0632 |  |  |  |  |  |  |  |  |  |  |  |  |
| TRS | 0.6655 | 0.6988 | 0.3337 | 0.0966 | 0.0910 | 0.1039 | 0.0357 | 0.0575 |  |  |  |  |  |  |  |  |  |  |  |
| TRL | 0.6117 | 0.6374 | 0.3246 | 0.0948 | 0.0904 | 0.1048 | 0.0194 | 0.0396 | 0.0138 |  |  |  |  |  |  |  |  |  |  |
| SV1 | 0.6310 | 0.6597 | 0.3227 | 0.1088 | 0.1060 | 0.1201 | 0.0372 | 0.0384 | 0.0103 | 0.0109 |  |  |  |  |  |  |  |  |  |
| SV2 | 0.6036 | 0.6395 | 0.2731 | 0.0959 | 0.0862 | 0.1026 | 0.0399 | 0.0566 | 0.0272 | 0.0300 | 0.0191 |  |  |  |  |  |  |  |  |
| SV3 | 0.6666 | 0.6993 | 0.3363 | 0.0957 | 0.0975 | 0.0994 | 0.0192 | 0.0433 | 0.0130 | 0.0109 | 0.0000 | 0.0106 |  |  |  |  |  |  |  |
| SV4 | 0.6292 | 0.6655 | 0.2800 | 0.1103 | 0.1121 | 0.1178 | 0.0436 | 0.0353 | 0.0031 | 0.0258 | 0.0066 | 0.0188 | 0.0011 |  |  |  |  |  |  |
| PSW | 0.6215 | 0.6489 | 0.3303 | 0.0939 | 0.0955 | 0.0960 | 0.0464 | 0.0848 | 0.0233 | 0.0269 | 0.0272 | 0.0395 | 0.0124 | 0.0293 |  |  |  |  |  |
| PSE | 0.6052 | 0.6273 | 0.3382 | 0.0872 | 0.0861 | 0.0915 | 0.0330 | 0.0752 | 0.0269 | 0.0238 | 0.0241 | 0.0276 | 0.0067 | 0.0339 | 0.0020 |  |  |  |  |
| WS1 | 0.4760 | 0.4965 | 0.2275 | 0.0680 | 0.0728 | 0.0746 | 0.0353 | 0.0603 | 0.0211 | 0.0221 | 0.0156 | 0.0141 | 0.0122 | 0.0075 | 0.0175 | 0.0170 |  |  |  |
| WS2 | 0.5834 | 0.6011 | 0.3445 | 0.0935 | 0.0981 | 0.0988 | 0.0436 | 0.0793 | 0.0271 | 0.0315 | 0.0231 | 0.0326 | 0.0110 | 0.0330 | 0.0062 | 0.0000 | 0.0191 |  |  |
| MTR | 0.0444 | 0.0179 | 0.2228 | 0.6508 | 0.6659 | 0.6618 | 0.6172 | 0.6163 | 0.7284 | 0.6581 | 0.6765 | 0.6656 | 0.7288 | 0.6965 | 0.6695 | 0.6407 | 0.5150 | 0.6204 |  |
| MGA | 0.7380 | 0.7742 | 0.3696 | 0.2752 | 0.2915 | 0.2910 | 0.2259 | 0.1130 | 0.3088 | 0.2218 | 0.2504 | 0.2705 | 0.2704 | 0.2299 | 0.3017 | 0.2816 | 0.2104 | 0.2733 | 0.8604 |

^a^ The pairwise F_ST_ value estimated in Genepop 4.2 (multi locus estimates following standard ANOVA as in Weir and Cockerham (1984) with a minimum distance between samples of 0.0001 and 1000 permutations for Mantel test). Slightly negative F_ST_ values were set to zero inferring lack of population structure.

^b^ For explanation of sample codes see Table 1.

**Table S3**. Pairwise F_ST_ values for *Mytilus edulis* samples including 81 loci.

| **F_ST_**^a^ | UPE^b^ | NUU | KOB | ICE | LOF | TRO | SVA | SV4 | PSW | PSE | WS1 |
| --- | --- | --- | --- | --- | --- | --- | --- | --- | --- | --- | --- |
| NUU | 0.0111 |  |  |  |  |  |  |  |  |  |  |
| KOB | 0.0082 | 0.0000 |  |  |  |  |  |  |  |  |  |
| ICE | 0.0476 | 0.0476 | 0.0468 |  |  |  |  |  |  |  |  |
| LOF | 0.0779 | 0.0970 | 0.1037 | 0.0434 |  |  |  |  |  |  |  |
| TRO | 0.0939 | 0.0908 | 0.1001 | 0.0247 | 0.0000 |  |  |  |  |  |  |
| SVA | 0.0929 | 0.0966 | 0.0991 | 0.0297 | 0.0000 | 0.0063 |  |  |  |  |  |
| SV4 | 0.0722 | 0.1053 | 0.1093 | 0.0265 | 0.0000 | 0.0121 | 0.0000 |  |  |  |  |
| PSW | 0.0889 | 0.0964 | 0.0941 | 0.0403 | 0.0151 | 0.0131 | 0.0171 | 0.0116 |  |  |  |
| PSE | 0.0898 | 0.0881 | 0.0902 | 0.0272 | 0.0190 | 0.0127 | 0.0099 | 0.0176 | 0.0034 |  |  |
| WS1 | 0.0824 | 0.0800 | 0.0805 | 0.0300 | 0.0000 | 0.0068 | 0.0087 | 0.0052 | 0.0098 | 0.0004 |  |
| WS2 | 0.0995 | 0.0958 | 0.0970 | 0.0377 | 0.0194 | 0.0170 | 0.0116 | 0.0141 | 0.0064 | 0.0000 | 0.0011 |

^a^ The pairwise F_ST_ value estimated in Genepop 4.2 (multi locus estimates following standard ANOVA as in Weir & Cockerham (1984) with a minimum distance between samples of 0.0001 and 1000 permutations for Mantel test). Slightly negative F_ST_ values were set to zero inferring lack of population structure.

^b^ For explanation of sample codes see Table 1. Further codes: NUU comprise of NUS and NUL. TRO comprise of TRS and TRL and SVA comprise of SV1, SV2 and SV3.

**Table S4**. Pairwise F_ST_ values for *Mytilus edulis* samples not including six outlier loci.

| **F_ST_**^a^ | UPE^b^ | NUU | KOB | ICE | LOF | TRO | SVA | SV4 | PSW | PSE | WS1 |
| --- | --- | --- | --- | --- | --- | --- | --- | --- | --- | --- | --- |
| NUU | 0.0070 |  |  |  |  |  |  |  |  |  |  |
| KOB | 0.0017 | 0.0000 |  |  |  |  |  |  |  |  |  |
| ICE | 0.0337 | 0.0413 | 0.0301 |  |  |  |  |  |  |  |  |
| LOF | 0.0233 | 0.0571 | 0.0512 | 0.0352 |  |  |  |  |  |  |  |
| TRO | 0.0517 | 0.0566 | 0.0513 | 0.0160 | 0.0021 |  |  |  |  |  |  |
| SVA | 0.0581 | 0.0668 | 0.0587 | 0.0224 | 0.0000 | 0.0064 |  |  |  |  |  |
| SV4 | 0.0249 | 0.0605 | 0.0448 | 0.0104 | 0.0000 | 0.0093 | 0.0000 |  |  |  |  |
| PSW | 0.0561 | 0.0712 | 0.0588 | 0.0327 | 0.0173 | 0.0106 | 0.0175 | 0.0090 |  |  |  |
| PSE | 0.0548 | 0.0591 | 0.0505 | 0.0198 | 0.0229 | 0.0124 | 0.0119 | 0.0113 | 0.0032 |  |  |
| WS1 | 0.0459 | 0.0483 | 0.0383 | 0.0222 | 0.0001 | 0.0067 | 0.0098 | 0.0000 | 0.0090 | 0.0000 |  |
| WS2 | 0.0695 | 0.0716 | 0.0624 | 0.0320 | 0.0228 | 0.0172 | 0.0131 | 0.0079 | 0.0069 | 0.0000 | 0.0009 |

^a^ The pairwise F_ST_ value determined in Genepop 4.2 (multi locus estimates following standard ANOVA as in Weir and Cockerham (1984) with a minimum distance between samples of 0.0001 and 1000 permutations for Mantel test). Slightly negative F_ST_ values were set to zero inferring lack of population structure.

^b^ For explanation of sample codes see Table 1. Further codes: NUU comprise of NUS and NUL. TRO comprise of TRS and TRL and SVA comprise of SV1, SV2 and SV3.

**Table S5**. Pairwise F_ST_ values for *Mytilus edulis* samples only including the six outlier loci.

| **F_ST_**^a^ | UPE^b^ | NUU | KOB | ICE | LOF | TRO | SVA | SV4 | PSW | PSE | WS1 |
| --- | --- | --- | --- | --- | --- | --- | --- | --- | --- | --- | --- |
| NUU | 0.0474 |  |  |  |  |  |  |  |  |  |  |
| KOB | 0.0695 | 0.0115 |  |  |  |  |  |  |  |  |  |
| ICE | 0.1668 | 0.1093 | 0.1983 |  |  |  |  |  |  |  |  |
| LOF | 0.3984 | 0.3595 | 0.4448 | 0.1228 |  |  |  |  |  |  |  |
| TRO | 0.4216 | 0.3716 | 0.4743 | 0.1261 | 0.0000 |  |  |  |  |  |  |
| SVA | 0.3553 | 0.3172 | 0.3907 | 0.1063 | 0.0000 | 0.0057 |  |  |  |  |  |
| SV4 | 0.3481 | 0.3802 | 0.4837 | 0.1624 | 0.0031 | 0.0486 | 0.0679 |  |  |  |  |
| PSW | 0.3238 | 0.2796 | 0.3489 | 0.1142 | 0.0000 | 0.0447 | 0.0130 | 0.0393 |  |  |  |
| PSE | 0.3542 | 0.3017 | 0.3781 | 0.1045 | 0.0000 | 0.0155 | 0.0000 | 0.0896 | 0.0058 |  |  |
| WS1 | 0.3651 | 0.3173 | 0.3896 | 0.1145 | 0.0000 | 0.0075 | 0.0000 | 0.1102 | 0.0184 | 0.0096 |  |
| WS2 | 0.3332 | 0.2847 | 0.3548 | 0.0991 | 0.0000 | 0.0149 | 0.0000 | 0.0804 | 0.0001 | 0.0000 | 0.0036 |

^a^ The pairwise F_ST_ value determined in Genepop 4.2 (multi locus estimates following standard ANOVA as in Weir and Cockerham (1984) with a minimum distance between samples of 0.0001 and 1000 permutations for Mantel test). Slightly negative F_ST_ values were set to zero inferring lack of population structure.

^b^ For explanation of sample codes see Table 1. Further codes: NUU comprise of NUS and NUL. TRO comprise of TRS and TRL and SVA comprise of SV1, SV2 and SV3.

**Literature cited**

Weir, B. S. and C. C. Cockerham. 1984. Estimating F-Statistics for the analysis of population-structure. Evolution **38**:1358-1370.

Zbawicka, M., A. Drywa, B. Smietanka, and R. Wenne. 2012. Identification and validation of novel SNP markers in European populations of marine *Mytilus* mussels. Marine Biology **159**:1347-1362.

Zbawicka, M., T. Sanko, J. Strand, and R. Wenne. 2014. New SNP markers reveal largely concordant clinal variation across the hybrid zone between *Mytilus* spp. in the Baltic Sea. Aquatic Biology **21**:25-36.
